# Selection of plant growth-promoting bacteria in sweet sorghum (*Sorghum bicolor *(L.) Moench) under the effects of salinity

**DOI:** 10.1186/1753-6561-8-S4-P113

**Published:** 2014-10-01

**Authors:** Marta Amâncio do Nascimento, Carlos Vinicius do Nascimento, Jadson Emanuel Antunes, Márcia do Vale Figueiredo, José Nildo Tabosa, Cosme Rafael Martínez

**Affiliations:** 1 IPA Instituto Agronômico de Pernambuco - Av. Gen. San Martin, 1371, Bongi, 50761-000, Recife-PE, Brazil; 2 Pós-Graduação em Biotecnologia - UFPB - RENORBIO, Brazil; 3 UFPB Universidade Federal da Paraíba, Departamento de Química - Cidade Universitária, 58051-900, João Pessoa-PB, Brazil

## Background

Sorghum, a grassy non-halophyte, it is both drought and salinity tolerant, and is considered a promising crop for semiarid regions. During the last 20 years, its culture has expanded in Brazilian production by 780%, reaching 1,928,970 tons in 2009 [1]. Salinization of the soil and lack of rain are increasing constraints in semiarid regions. They predominate in northeastern Brazil. This has helped limit the production of plant biomass, which is the basis of agricultural activity. The advantages of sorghum in these conditions can be improved still further by using plant growth-promoting bacteria (PGPB) for the biological processes of nitrogen fixation, and hormone production, as well as others [2]. Endophytic bacteria can secrete up to one half of the nitrogen they fix, and plants may then assimilate the nitrogen efficiently [3]. Inoculation with *Azotobacter chroococcum *has been shown to attenuate stress in maize grown in saline soil [4]. In this study, we evaluated the action of PGPB on a "Wray" saccharine variety with low salinity tolerance.

## Methods

The experiment used a factorial arrangement (2x10) with two blocks. The salinity conditions were 8 and 75 mM NaCl (Hoagland solution - SH). The isolates tested were *Herbaspirillum seropedicae *(Hs08 and Hs09), *Burkholderia vietnamiensis *(Bv12), *B. phymatum *(Bp16), *H. seropedicae*/ *Burkholderia *spp. (Hs/B30), and *Burkholderia *spp. (B62). All were tested with inorganic nitrogen (N) at 4 mM, and un-inoculated treatments of inorganic N at 2, 4, 8, and 16 mM. The bacteria were isolated in the state of Paraíba - Brazil. Disinfected seeds were sown in sterile sand + SH (pH 6.5), and inoculated with standardized bacterial suspensions (*A*_600 _= 0.500). The plants were grown (35 days) in a growth chamber under controlled conditions.

## Results and conclusions

Compared to the controls (4 mM N), significant increases in shoot biomass SB (3x), transpiration T (3x), and root biomass RB (2x) were observed (when N increased under non-saline conditions). Plant height PH response was lower (1.2x). However, under saline conditions, the inorganic N induced significant and varied reductions (-0.2x in PH, -0.3x for SB, -0.5x for RB and -0.4x for T). We found specific relationships between the bacteria used and both the plant's growth, and sensitivity to salinity. The isolate Hs09 in non-saline conditions provided an T and an SB equivalent to those obtained at 8 mM N. However, the greatest benefits were seen for PH and RB. Under saline conditions, Bp16 induced 2x higher SB, BR, and T, and 1.2x higher PH than in the control. Inoculations with Hs08, and Bp16 significantly improved SB, T, and thus the Wray salinity tolerance. Perhaps the action of hormones, improved nutritional status, or osmo-regulation induced by the bacteria-plant interaction may have contributed to this performance [2-4]. The stability of the PGPB action for Hs09, Hs08, and Bp16, and their attenuation of stress (Hs08 and Bp16) must be tested in other varieties, and saline soils to validate the use of this biotechnological factor for sustainable production of sorghum in saline environments.
